# miR-192, a prognostic indicator, targets the SLC39A6/SNAIL pathway to reduce tumor metastasis in human hepatocellular carcinoma

**DOI:** 10.18632/oncotarget.6603

**Published:** 2015-12-14

**Authors:** Junwei Lian, Ying Jing, Qiongzhu Dong, Lin Huan, Di Chen, Chunyang Bao, Qifeng Wang, Fangyu Zhao, Jinjun Li, Ming Yao, Lunxiu Qin, Linhui Liang, Xianghuo He

**Affiliations:** ^1^ State Key Laboratory of Oncogenes and Related Genes, Shanghai Cancer Institute, Renji Hospital, Shanghai Jiao Tong University School of Medicine, Shanghai 200032, China; ^2^ Institutes of Biomedical Sciences, Shanghai Medical College, Fudan University, Shanghai 200032, China; ^3^ Department of General Surgery, Huashan Hospital, Fudan University, Shanghai 200032, China; ^4^ Fudan University Shanghai Cancer Center, Shanghai Medical College, Fudan University, Shanghai 200032, China; ^5^ Liver Cancer Institute, Zhongshan Hospital, Fudan University, Shanghai 200032, China

**Keywords:** miR-192, SLC39A6, metastasis, prognosis, hepatocellular carcinoma

## Abstract

Metastasis is one of the causes of cancer death. Functions and mechanisms of microRNAs (miRNAs) involved in hepatocellular carcinoma (HCC) metastasis are largely unknown. Here, a miRNA microarray analysis was performed in MHCC-97L, MHCC-97H and HCC-LM3 cells with gradually increasing metastatic potential to disclose crucial miRNAs involved in HCC metastasis. miR-192 expression decreased and negatively correlated with vascular invasion in HCC specimens. Gain and loss of function studies revealed that miR-192 significantly suppressed metastasis of HCC cells *in vitro* and *in vivo*. Solute carrier family 39 member 6 (SLC39A6) was identified as a direct and functional target of miR-192. In addition, SLC39A6 negatively correlated with miR-192 in HCC samples and promoted HCC cell migration and invasion. Moreover, miR-192 decreased SLC39A6 expression, subsequently downregulating SNAIL and upregulating E-cadherin expression. Suppression of migration and invasion caused by miR-192 overexpression was alleviated by exogenous Snail expression. Intriguingly, lower miR-192 expression and higher SLC39A6 expression significantly contributed to poorer outcomes in HCC patients. Multivariate analysis indicated that miR-192 was an independent and significant predictor of HCC patient overall survival. In conclusion, we newly determined that miR-192 targeted the SLC39A6/SNAIL pathway to reduce tumor metastasis in HCC cells. This axis provided insights into the mechanism underlying miRNA regulation of HCC metastasis and a novel therapeutic target for HCC treatment.

## INTRODUCTION

Primary liver cancer, particularly hepatocellular carcinoma (HCC), continues to be a growing global health problem and is the third most common cause of cancer-related deaths worldwide, accounting for over 800,000 deaths per year [1, 2]. In China, the mortality rate of HCC is the second highest across the country and the 5-year survival rate is below 5% [3]. Among the causes of cancer-related deaths, metastasis accounts for 90% of cancer mortality [4–6]. Although a number of reports have demonstrated that multiple signaling pathways are involved in tumor metastasis, the molecular mechanisms governing the metastatic cascades of HCC are complex, and our current knowledge regarding these mechanisms remains limited [4, 7].

MicroRNAs (miRNAs) are post-transcriptional regulators of gene expression that bind primarily to complementary sequences in the 3′ untranslated region (UTR) of their target mRNAs and cause translational repression or mRNA degradation of target genes [8, 9]. In human cells, miRNAs are estimated to regulate more than one-third of human genes and to further influence the majority of genetic pathways [10]. miRNAs can function as oncogenes or tumor suppressors, and their dysfunctions are involved in the development and progression of various human cancers [11, 12]. Moreover, miRNA expression profiles or signatures in cancer can be utilized for early diagnosis, molecular subtyping, and patient outcome prediction [13, 14]. However, the functions and mechanisms of miRNAs involved in HCC metastasis are largely unknown.

In this study, we performed miRNA arrays in three stepwise metastatic HCC cell lines (MHCC-97L, MHCC-97H, HCC-LM3) to reveal miRNAs that drive HCC invasion and metastasis. MHCC-97L, MHCC-97H, HCC-LM3 are derived from one parental cell line, MHCC-97, and possess stepwise increases in metastatic potential [15, 16]. miRNAs that are differentially expressed in the three cell lines may directly regulate HCC metastasis. Among these differentially expressed miRNAs, miR-192 was often downregulated in HCC. Importantly, miR-192 was negatively associated with metastasis and poor prognosis and could be an independent indicator for HCC patient outcome. We further identified miR-192 as a metastasis suppressor of HCC and SLC39A6, which is an oncogene involved in different types of cancer [17–22], as a direct and functional target for miR-192 in HCC.

## RESULTS

### miR-192 was downregulated in metastatic HCC cell lines and HCC specimens

**Figure 1:**
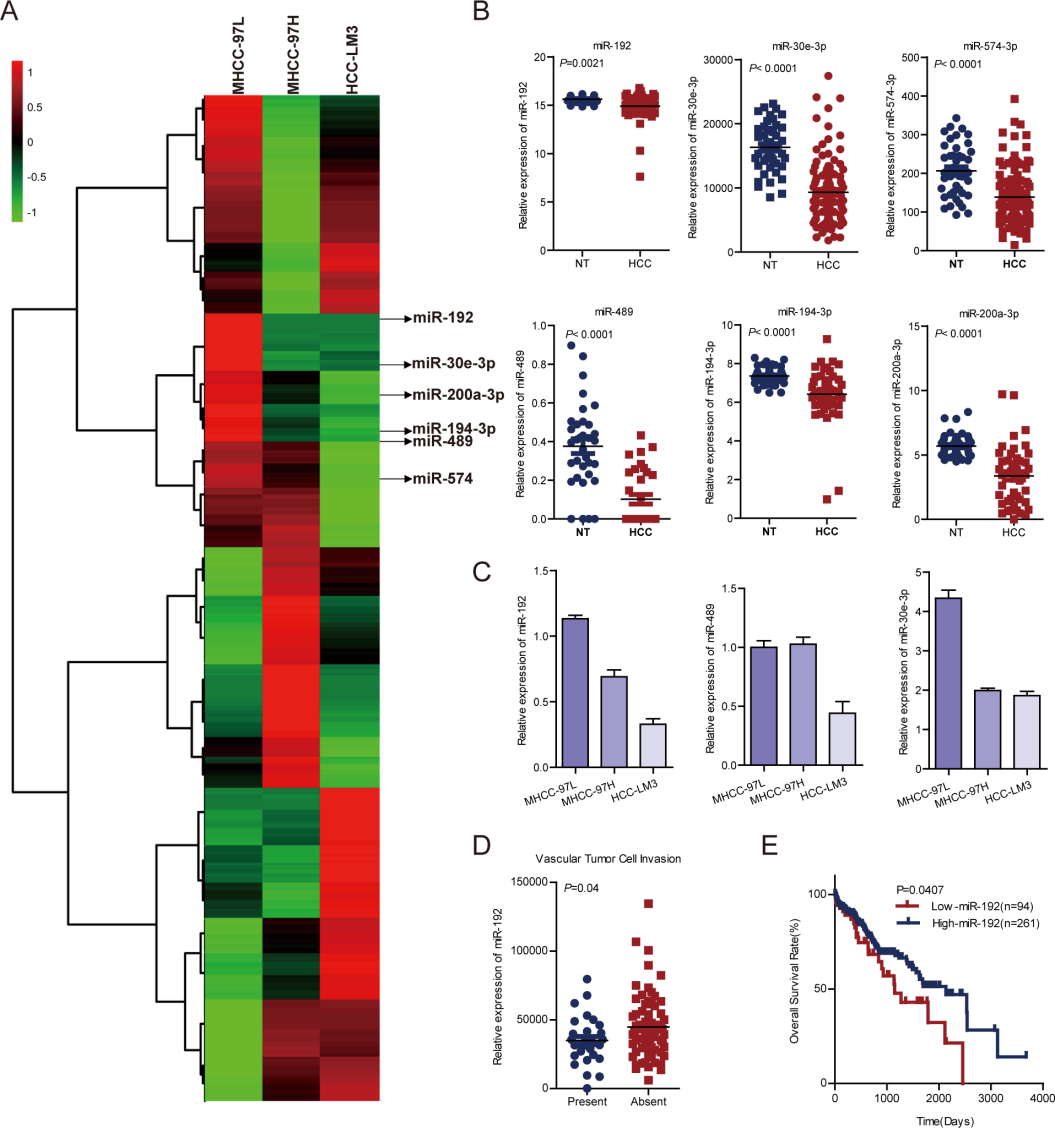
miR-192 was downregulated in metastatic HCC cell lines and HCC tissues (**A**) Heat map. miRNA microarray analyses were performed in MHCC-97L, MHCC-97H and HCC-LM3 cell lines. The green in the legend bar represented downregulation, and the red represented upregulation. (**B**) Analysis of miR-192, miR-30e-3p, miR-574–3p, miR-489, miR-194–3p and miR-200a-3p expression in TCGA dataset (paired Student's *t*-test). NT, non-tumor tissue; HCC, primary tumor. (**C**) Determination of miR-192, miR-30e and miR-489 expression levels in MHCC-97L, MHCC-97H and HCC-LM3 cell lines (mean ± SEM). (**D**) Downregulation of miR-192 expression in primary HCCs with vascular tumor cell invasion compared with those without (unpaired Student's *t*-test). (**E**) Kaplan-Meier analysis of miR-192 expression in HCC patient tissue samples in TCGA dataset. The point in the ROC curve with the highest absolute Youden index was used as the threshold to divide HCC patients into two groups (high vs. low).

To determine the roles of miRNAs in HCC metastasis, we performed miRNA microarrays in MHCC-97L, MHCC-97H and HCC-LM3 cells with gradually increasing metastatic potential [16] (Figure 1A). Among the differentially expressed miRNAs, 27 miRNAs were upregulated or downregulated in MHCC-97H and HCC-LM3 cells (Supplementary Table 1). Notably, six miRNAs (miR-489, miR-194–3p, miR-200a-3p, miR-30e-3p, miR-192, and miR-574–3p) were identically decreased in both our microarrays and The Cancer Genome Atlas (TCGA) dataset (Supplementary Table 1, Figure 1B). miR-194 and miR-200a-3p have been shown to suppress invasion and metastasis in HCC [24–26]. Next, we analyzed the effects of the other four miRNAs on HCC cell migration. The results showed that miR-574 did not affect HCC cell migration (Supplementary Figure 1A). Quantitative real-time PCR (q-PCR) was performed to detect the expression levels of miR-192, miR-489 and miR-30e-3p in MHCC-97L, MHCC-97H and HCC-LM3, and only miR-192 was downregulated in a stepwise fashion across the three cell lines (Figure 1C). Moreover, clinical significance analysis of TCGA data showed that miR-192 expression levels were lower in HCC patients with vascular cell invasion compared with HCC patients without vascular cell invasion (Figure 1D). Most importantly, patients with higher miR-192 expression levels had better overall survival rates than did patients with lower miR-192 expression levels (Figure 1E). Taken together, miR-192 expression decreased stepwise in HCC cells with gradually increasing metastatic potential and was downregulated in HCC samples, indicating that miR-192 might act as a metastasis suppressor in HCC.

### miR-192 suppressed HCC cell metastasis *in vitro* and *in vivo*

**Figure 2:**
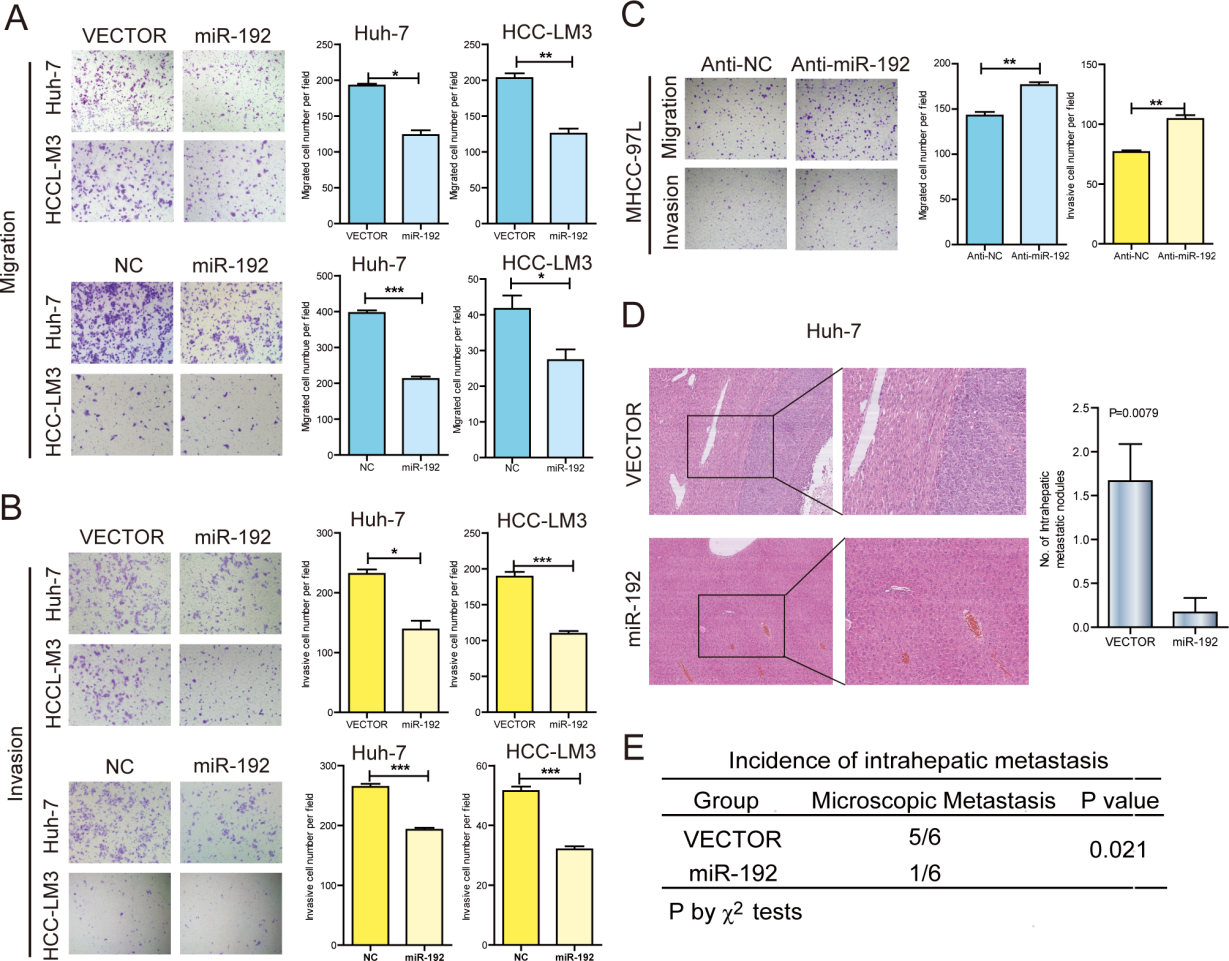
miR-192 suppressed HCC cell metastasis *in vitro* and *in vivo* (**A**) Representative results of the Transwell migration assays showing the effect of miR-192 expression on the migratory abilities of HCC-LM3 and Huh-7 cells (unpaired Student's *t*-test, mean ± SEM; *P < 0.05; **P < 0.01; ***P < 0.001). (**B**) Representative results of the Transwell invasion assays showing the effect of miR-192 expression on the invasive abilities of HCC-LM3 and Huh-7 cells (unpaired Student's *t*-test, mean ± SEM; *P < 0.05; **P < 0.01; ***P < 0.001). (**C**) Representative results of the Transwell migration and invasion assays showing the effect of miR-192 inhibition on the migratory and invasive abilities of MHCC-97L cells (unpaired Student's *t*-test, mean ± SEM; *P < 0.05; **P < 0.01; ***P < 0.001). (**D** and **E**) The effects of miR-192 expression on the *in vivo* metastatic abilities of Huh-7 cells in an orthotopic liver xenograft model of nude mice (*n =* 6) as determined by examination of mouse livers for microscopic nodules. Representative images of the histological examination of mouse livers for primary tumors and metastatic nodules from Huh-7-vector and Huh-7-miR-192 cells (D), and the incidence of intrahepatic metastasis in the two groups of this mouse model (E). Statistical analysis of the difference between groups was performed by a chi-square (χ^2^) test and unpaired Student's *t*-test, mean ± SEM; *P* < 0.05 was considered statistically significant.

To investigate the function of miR-192 in HCC, we first determined the intrinsic expression levels of miR-192 in seven HCC cell lines (Supplementary Figure 2A). HCC-LM3, Huh-7 and SK-Hep-1 cell lines were used to analyze the effects of miR-192 overexpression. miR-192 mimic transfection significantly inhibited HCC cell migration and invasion *in vitro* (Figure 2A and 2B, Supplementary Figure 2B), whereas miR-192 inhibitor transfection promoted HCC cell migration and invasion (Figure 2C). Moreover, stably overexpressed miR-192 (Supplementary Figure 2C) resulted in the suppression of migration and invasion in HCC-LM3 and Huh-7 cells (Figure 2A and 2B). Elevated miR-192 expression did not affect HCC cell growth (Supplementary Figure 2D). To further determine the effect of miR-192 on metastasis *in vivo*, Huh-7-miR-192 and Huh-7-vector stable cell lines were transplanted into the livers of nude mice. After six weeks, the mice were euthanized and the livers were fixed and examined. The numbers of intrahepatic metastatic nodules and the incidence of intrahepatic metastasis were significantly lower in the miR-192 group than were those in the vector group (Figure 2D and 2E). Altogether, miR-192 inhibited the invasion and metastasis of HCC cells *in vitro* and *in vivo*.

### SLC39A6 was a direct downstream target of miR-192 in HCC cells

**Figure 3:**
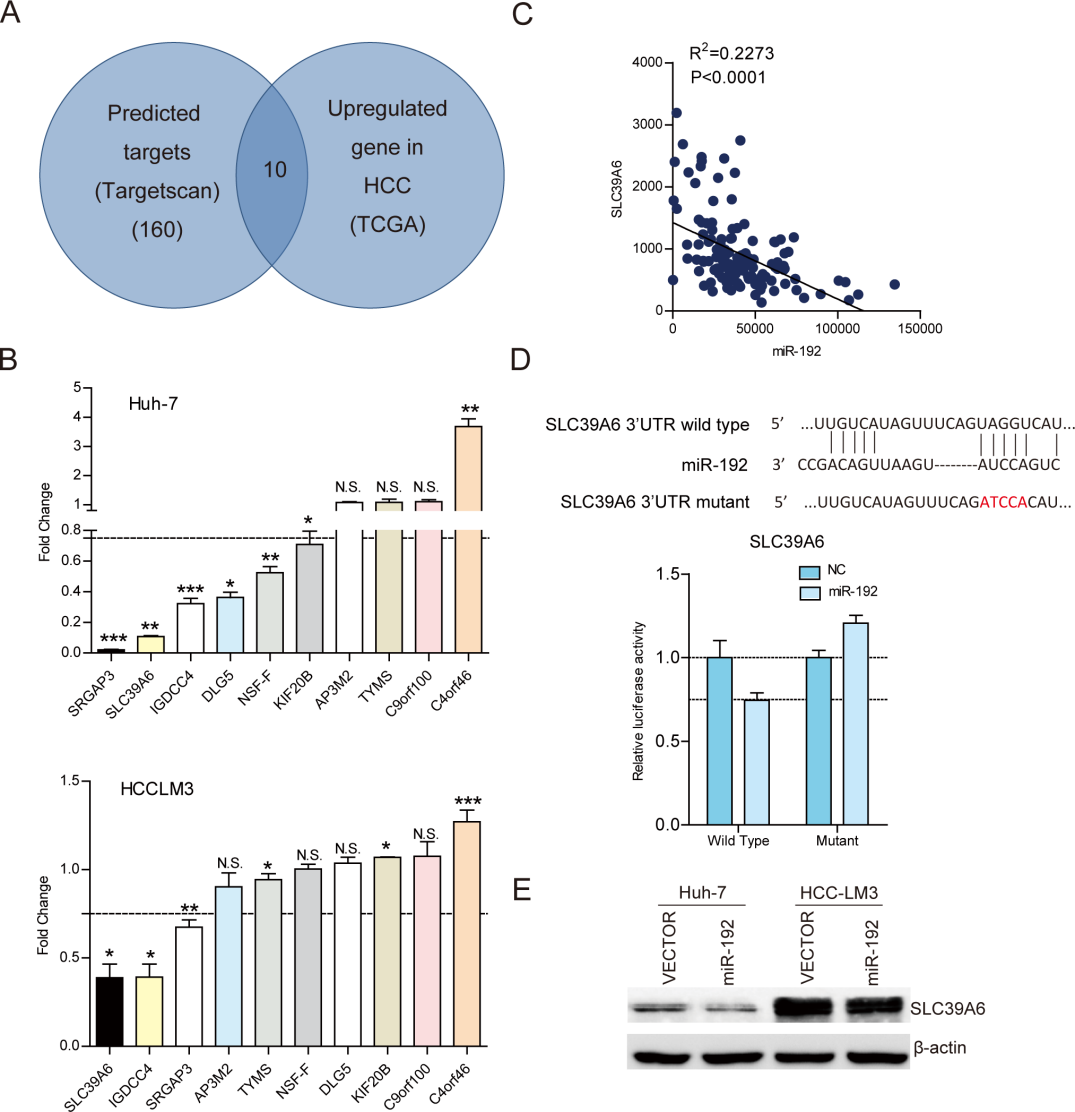
*SLC39A6* was a direct downstream target of miR-192 in HCC cells (**A**) Intersection of TargetScan predicted miR-192 targets and upregulated genes in TCGA dataset. (**B**) Determination of the expression levels of ten candidates in Huh-7 and HCC-LM3 cell lines by q-PCR. (**C**) Correlation analysis of miR-192 expression and *SLC39A6* expression in 130 HCC tissues in TCGA dataset. The correlation was analyzed by two-tailed Pearson's correlation test. (**D**) Top, miR-192 and its putative binding sequence in the *SLC39A6* 3′ UTR. The mutant miR-192 binding sequence was generated in the seed region. Bottom, examination of luciferase activity. Co-transfection of a wildtype or a mutant *SLC39A6* 3′UTR with miR-192 mimics into HEK-293T cells. Firefly luciferase activity was measured and standardized by *Renilla* luciferase activity. (**E**) Detection of *SLC39A6* downregulation due to miR-192 overexpression in Huh-7 and HCC-LM3 cells by immunoblotting.

To explore the molecular mechanisms by which miR-192 inhibited HCC cell metastasis, we predicted 160 potential targets of miR-192 in TargetScan (http://www.targetscan.org/). Interestingly, among these candidate targets, 10 were upregulated in the HCC tissues from TCGA dataset (Figure 3A). Then, we determined the expression of 10 candidates by q-PCR after miR-192 mimic transfection. Only three genes, *SLC39A6*, *IGDCC4* and *SRGAP3*, were downregulated after miR-192 transfection in two cell lines (Figure 3B). Correlation analysis between *SLC39A6*, *IGDCC4*, *SRGAP3* and miR-192 in TCGA dataset revealed that *SLC39A6* expression negatively correlated with miR-192 expression, while *IGDCC4* and *SRGAP3* expression did not (Figure 3C, Supplementary Figure 3A). The wild type and mutant 3′ UTRs of *SLC39A6* were cloned and inserted into a luciferase reporter vector. The luciferase activity of the wild type 3′ UTR of *SLC39A6* was downregulated in the presence of miR-192, while the luciferase activity of the mutant 3′ UTR of *SLC39A6* remained unchanged (Figure 3D). Moreover, the SLC39A6 protein level was downregulated in miR-192-overexpressing cells (Figure 3E). Taken together, these results indicated that *SLC39A6* was a direct downstream target of miR-192 in HCC cells.

### SLC39A6 promoted HCC cell migration and invasion

**Figure 4:**
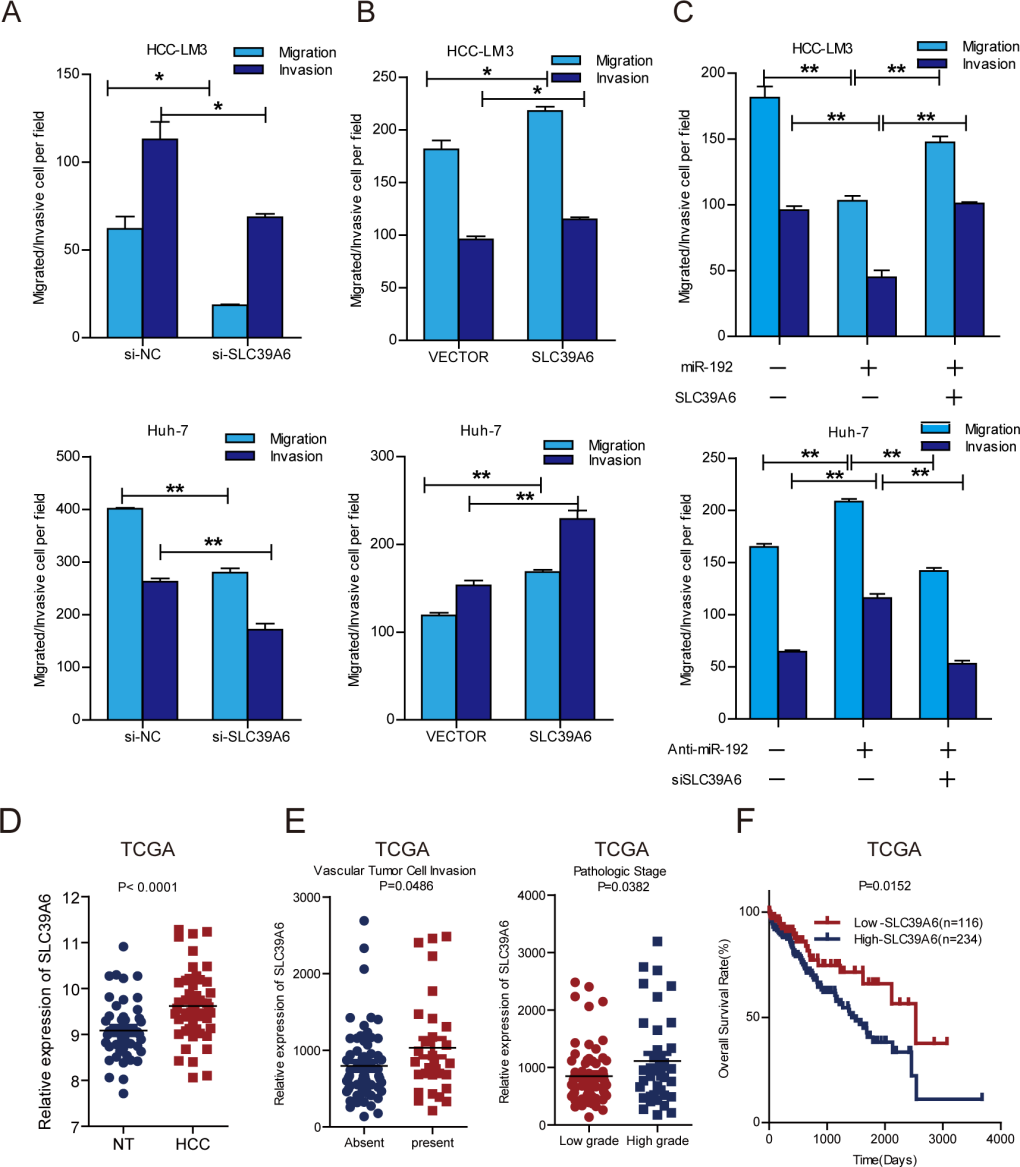
SLC39A6 promoted HCC cell migration and invasion (**A**) Transwell migration and invasion assays of HCC-LM3 and Huh-7 cells were performed after *SLC39A6* siRNA or NC (negative control) transfection (unpaired Student's *t*-test, mean ± SEM; *P < 0.05; **P < 0.01; ***P < 0.001). (**B**) Transwell migration and invasion assays of HCC-LM3 and Huh-7 cells were performed after *SLC39A6* overexpression. Empty vector (vector) was used as control (unpaired Student's *t*-test, mean ± SEM; *P < 0.05; **P < 0.01; ***P < 0.001). (**C**) Transwell assays for the SLC39A6-rescued migratory and invasive abilities of HCC-LM3 miR-192 overexpressing cells and *SLC39A6*-knockdown in miR-192 inhibitor transfected Huh-7 cells (unpaired Student's *t*-test, mean ± SEM; *P < 0.05; **P < 0.01; ***P < 0.001). (**D**) Analysis of *SLC39A6* expression in TCGA dataset (paired Student's *t*-test). NT, non-tumor tissue; HCC, primary tumor. (**E**) Left, upregulation of *SLC39A6* in primary HCCs with vascular tumor cell invasion compared with those without (unpaired Student's *t*-test). Right, upregulation of *SLC39A6* in primary HCCs in patients with high-grade (grades III–IV) tumors compared to those with low-grade (grades I–II) tumors (unpaired Student's *t*-test). (**F**) Kaplan-Meier analysis of *SLC39A6* expression in HCC patients in TCGA dataset. The point in the ROC curve with the highest absolute value of the Youden index was used as the threshold to divide the HCC patients into two groups (high vs. low).

SLC39A6, also named LIV-1, is a zinc transporter that regulates the invasion and metastasis of pancreas, breast and prostate cancers [20, 22, 27]. It was reported that SLC39A6 expression is negative correlated with E-cadherin, thus might participated in EMT in HCC [28]. However, the function and mechanisms of SLC39A6 in HCC metastasis has remained unknown. Therefore, we investigated the function of SLC39A6 in HCC cell migration and invasion using siRNA against *SLC39A6* (Supplementary Figure 3B and 3C) and overexpression of *SLC39A6* (Supplementary Figure 3D) in HCC cells. The results showed that *SLC39A6* knockdown significantly decreased the migration and invasion of HCC-LM3 and Huh-7 cells (Figure 4A) and that *SLC39A6* overexpression remarkably enhanced the migration and invasion of HCC cells (Figure 4B). The restoration of *SLC39A6* expression in cells stably expressing miR-192 blocked the miR-192-induced suppression of migration and invasion (Figure 4C, Supplementary Figure 3E) and knockdown of SLC39A6 abolished migration and invasion elevation in miR-192 inhibited cells (Figure 4C), indicating that SLC39A6 mediated the suppressive effects of miR-192 on HCC migration and invasion. Next, we examined the expression of *SLC39A6* in HCC samples from TCGA dataset. The results showed that *SLC39A6* expression was significantly upregulated in HCC tissues compared to that in the non-cancerous liver tissues (Figure 4D) and that its expression was higher in HCC samples with vascular cell invasion and high pathological grade (Figure 4E). Intriguingly, higher *SLC39A6* expression levels predicted poor outcomes for HCC patients (Figure 4F). Taken together, these results indicated that SLC39A6 promoted HCC cell migration and invasion, was negatively correlated with overall survival of HCC patients and functioned as a downstream mediator of miR-192.

### miR-192 decreased SNAIL expression by targeting SLC39A6 in HCC cells

**Figure 5:**
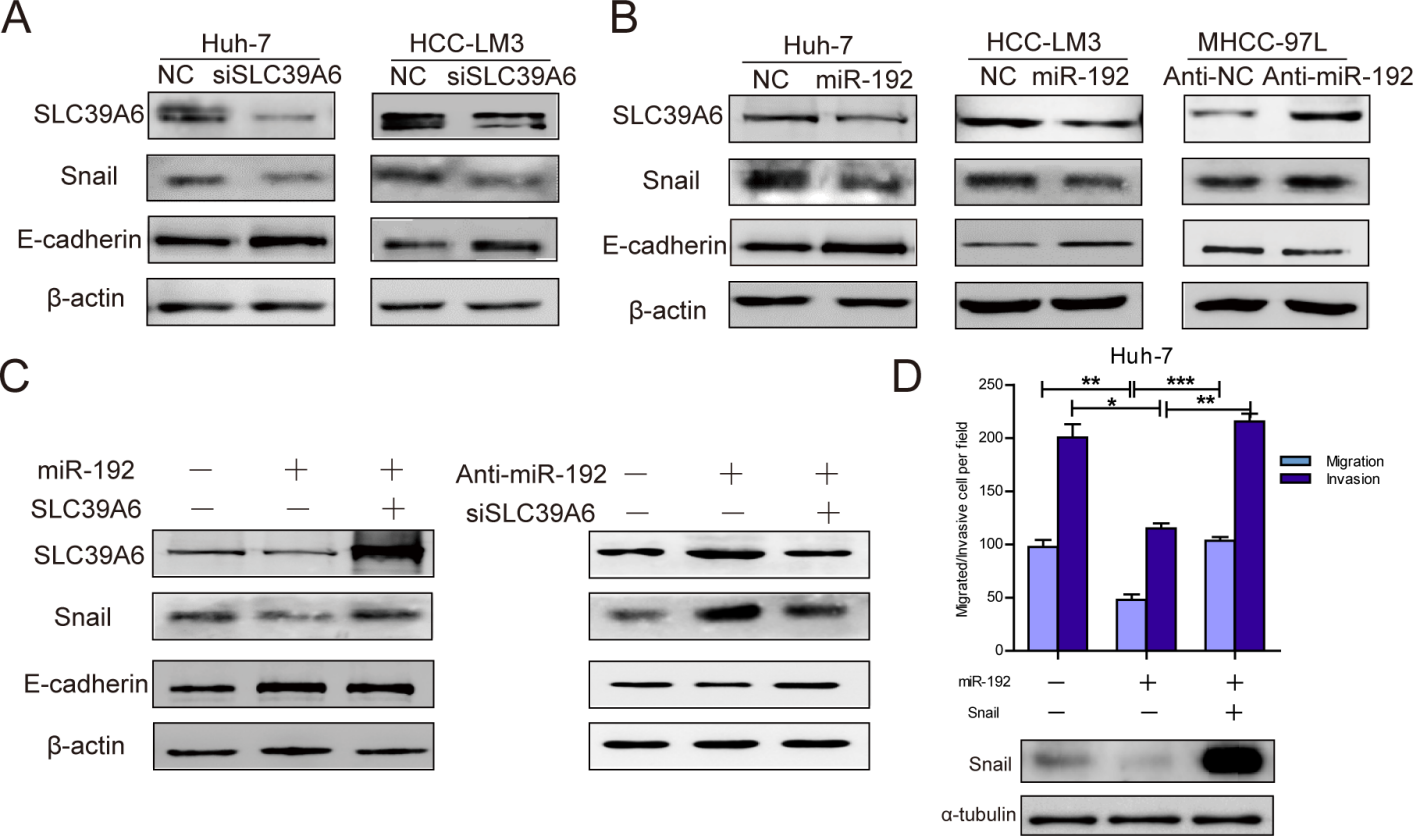
miR-192 inhibited SLC39A6/SNAIL/E-cadherin pathways in HCC cells (**A**) *SLC39A6*-specific siRNA transfection into Huh-7 and HCC-LM3 cells resulted in downregulated SNAIL expression and upregulated E-cadherin expression. (**B**) miR-192 mimic transfection into Huh-7 and HCC-LM3 cells decreased SNAIL protein levels and increased E-cadherin protein levels (left, middle). miR-192 inhibitor transfection into MHCC-97L cells upregulated SNAIL expression and downregulated E-cadherin expression (right). (**C**) Left, re-expressing *SLC39A6* in miR-192-overexpressing cells recovered SNAIL and E-cadherin protein levels; Right, downregulation of *SLC39A6* in miR-192 inhibited cells reversed the SNAIL and E-cadherin protein alteration. (**D**) Top, transwell migration and invasion assays showing the effect of ectopic SNAIL expression in Huh-7 cells stably expressing miR-192 (unpaired Student's *t*-test, mean ± SEM; *P < 0.05; **P < 0.01; ***P < 0.001); Bottom, determination of SNAIL expression in designated cells by Western blot.

SLC39A6 has been reported to regulate SNAIL and E-cadherin expression in breast cancer [21]. We found that *SLC39A6* knockdown significantly downregulated SNAIL expression and upregulated E-cadherin expression in HCC cells (Figure 5A). Remarkably, SNAIL expression decreased and E-cadherin expression increased following *SLC39A6* downregulation in miR-192 mimic-transfected cells (Figure 5B). Consistent with these results, miR-192 inhibitor transfection increased SLC39A6 protein levels and altered SNAIL and E-cadherin expression levels (Figure 5B). Furthermore, re-expression of *SLC39A6* in cells stably expressing miR-192 reversed SNAIL and E-cadherin expression levels alteration induced by miR-192 and knockdown of *SLC39A6* after miR-192 inhibitor transfection also abrogated protein change of SNAIL and E-cadherin (Figure 5C). These results suggested that miR-192 might regulate SNAIL and E-cadherin expression by targeting *SLC39A6*. Moreover, restoration of SNAIL expression reversed the miR-192-induced suppression of HCC cell migration and invasion (Figure 5D). These data suggested that miR-192 targeted the SLC39A6/SNAIL pathway to suppress HCC cell migration and invasion.

### Prognostic significance of miR-192 in HCC patients

**Figure 6:**
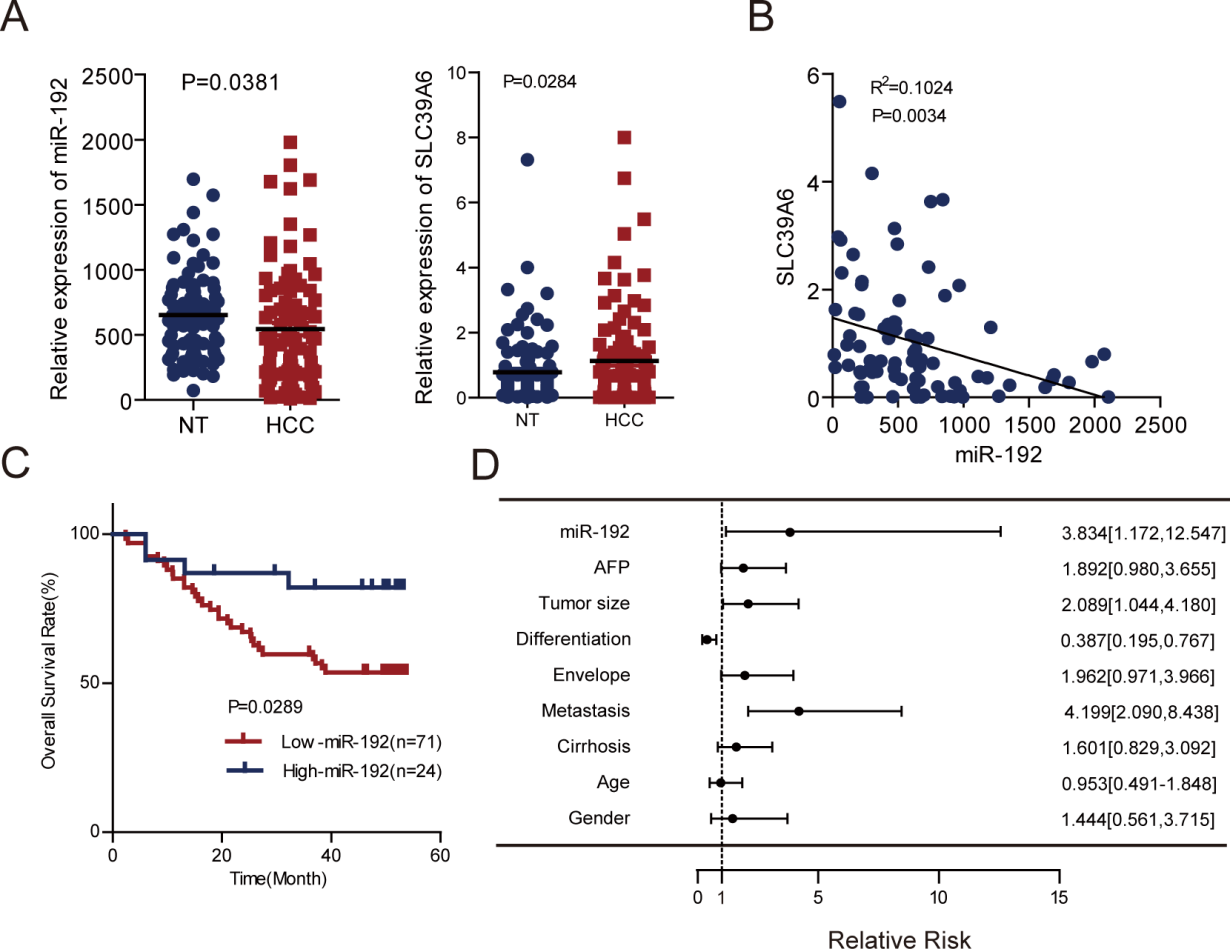
miR-192 was an independent predictor for HCC patient outcomes (**A**) Left, determination of miR-192 expression levels by q-PCR in an independent cohort of 101 paired HCCs and adjacent non-tumor tissues (paired Student's *t*-test). Right, determination of *SLC39A6* gene expression levels by q-PCR in an independent cohort of 101 paired HCCs and adjacent non-tumor tissues with complementary DNA (cDNA) (paired Student's *t*-test). (**B**) Correlation analysis of miR-192 and *SLC39A6* expression in 82 HCC primary tissues with both miR-192 and *SLC39A6* expression. The correlation was analyzed by two-tailed Pearson's correlation test. (**C**) Kaplan-Meier analysis of 95 paired HCC tissues preserved in our lab revealed that the high-miR-192 group had better overall survival rates. The point in the ROC curve with the highest absolute value of the Youden index was used as the threshold to divide HCC patients into two groups. (**D**) Forest plot of miR-192, AFP, tumor size, differentiation, envelope, metastasis, cirrhosis, age and gender as univariate predictors of overall survival in HCC patients.

To further evaluate the clinical significance of the miR-192/SLC39A6 axis in HCC, we determined miR-192 and *SLC39A6* expression levels in another independent cohort of tumors and adjacent non-tumor tissues from 101 HCC patients. Consistent with the expression pattern in TCGA, miR-192 expression was also downregulated, whereas *SLC39A6* expression was upregulated in the HCC samples (Figure 6A). miR-192 expression negatively correlated with *SLC39A6* expression (Figure 6B), which suggested that upregulation of *SLC39A6* might be due to downregulation of miR-192 in HCC. Importantly, HCC patients with higher miR-192 expression levels had better overall survival than the group with lower miR-192 expression levels (Figure 6C). Univariate analyses using the Cox hazard regression model identified low miR-192 expression, tumor size, differentiation and metastasis as prognostic indicators of overall survival for HCC patients (Figure 6D, Table 1). Multivariate analysis further demonstrated that low miR-192 expression was an independent and significant risk factor of overall survival for HCC patients (hazard ratio, 3.739; 95% CI, 1.127–12.407; *P* = 0.031; Table 2). These results revealed a significant contribution of higher miR-192 expression to better HCC patient outcomes and indicated that miR-192 was an independent and significant prognostic factor for HCC patients.

**Table 1:** Univariate analyses of factors associated with overall survial rate in HCC patients

Factors	OS Relative risk	(95% CI)	*P* value
Gender (male vs. female)	1.444	0.561–3.715	0.446
Age (> 50 vs. < = 50)	0.953	0.491–1.848	0.886
Cirrhosis	1.601	0.829–3.092	0.161
Metastasis	4.199	2.090–8.438	0.000
Envelope (Absent vs. Present)	1.962	0.971–3.966	0.060
Differeniation (I II vs. III IV)	0.387	0.195–0.767	0.007
Tumore size (> 5cm vs. < = 5cm)	2.089	1.044–4.180	0.038
AFP (ng/ml) (> 20 vs. < = 20)	1.892	0.980–3.655	0.058
miR-192 expression (low vs. high)	3.834	1.172–12.547	0.026

Univariate analysis was performed by the Cox proportional hazards regression model.

CI: confidential interval; OS:overall survival.

**Table 2:** Multivariate analyses of factors associated with overall survival rate in HCC patients

Variables	HR	95% CI	*P* value
Metastasis	3.666	1.689–7.961	0.001
Differentiation (I II vs. III IV)	0.729	0.338–1.570	0.419
Tumore size (>5cm vs. < = 5cm)	1.651	0.810–3.366	0.167
miR-192 expression (low vs. high)	3.739	1.127–12.407	0.031

Cox multivariate proportional hazard regression was adopted in multivariate analyses.

HR: hazard ratio; CI: confidential interval.

## DISCUSSION

Invasion and metastasis are hallmarks of cancer [28] and crucial processes in cancer progression [4]. In this study, we systematically profiled miRNAs whose expression levels significantly changed in MHCC-97L, MHCC-97H and HCC-LM3 cell lines. These cell lines were derived from the same parental cell line but had stepwise increases in metastatic potential [29], which made them the ideal model for discovering crucial molecules involved in HCC metastasis, such as MMPs [16]. This unbiased interrogation of this cell model identified miR-192 as a potential metastasis suppressor in HCC. Notably, our screen also revealed other miRNAs as promising metastatic-related candidates in HCC, including well-known metastasis suppressor miRNAs [25, 30].

miR-192 was previously reported to inhibit the liver metastasis of colon cancer through targeting Bcl-2, Zeb-2, and VEGFA [31], to suppress tumor progression in renal cell carcinoma [32], to inhibit cell proliferation and induce apoptosis in lung cancer [33], and to be a biomarker of distant metastasis in gastric cancer [34]. However, the function of miR-192 in HCC has remained unexplored. In this study, we found that miR-192 expression decreased stepwise in cell lines with gradually increasing metastatic potential and was downregulated in HCC tissues of 101 independent pairs of HCC patients and TCGA dataset. Moreover, miR-192 significantly suppressed HCC cell invasion and metastasis *in vitro* and *in vivo*. These findings confirmed that miR-192 was an essential suppressor of metastasis in HCC. Importantly, better overall survival rates of HCC patients correlated with higher miR-192 expression levels. Multivariate analysis indicated that low miR-192 expression was a distinguishing and independent risk factor of HCC patients, with a higher hazard ratio than any other variables. Thus, miR-192 could act as a useful indicator for HCC outcomes.

In addition, *SLC39A6* was identified as a direct downstream target of miR-192 in HCC cells. SLC39A6 belongs to a subfamily of proteins that have structural characteristics of zinc transporters. SLC39A6 has been shown to regulate the invasion and metastasis of breast and prostate cancers [22, 27, 35]. Our study is the first time to demonstrate that *SLC39A6* expression promoted HCC cell migration and invasion. *SLC39A6* was upregulated in HCC samples and positively correlated with vascular tumor cell invasion and pathological stage. The overall survival of HCC patients was better in patients with high *SLC39A6* expression than in patients with low *SLC39A6* expression. These results demonstrated that *SLC39A6* played an important role in HCC metastasis. Moreover, *SLC39A6* expression was inversely associated with miR-192 expression in two independent HCC samples, which suggested that *SLC39A6* upregulation in HCC might be caused by miR-192 downregulation. SLC39A6 is essential for the nuclear localization of the zinc-finger protein SNAIL [21], a master regulator of cancer metastasis [36]. We also found that miR-192 decreased SNAIL expression by downregulating *SLC39A6* expression in HCC cells. The expression of E-cadherin, a downstream effector of SNAIL [37], was upregulated by miR-192. miR-192 inhibited HCC cell migration and invasion by downregulating *SLC39A6* expression and inactivating the SLC39A6/SNAIL signaling pathway. Restoration of SNAIL expression could antagonize the inhibition of miR-192.

In conclusion, we newly identified miR-192/SLC39A6/SNAIL as an important signaling pathway that governed HCC metastasis. miR-192 and SLC39A6 might be useful indicators for HCC patient outcomes, and the miR-192/SLC39A6/SNAIL pathway might be a promising therapeutic target for HCC treatment.

## MATERIALS AND METHODS

### HCC specimens

HCC primary tumors and adjacent non-tumor liver tissues (3 cm from the tumor) were obtained from the surgical specimen archives of the Zhongshan Hospital, Shanghai, China. The participants that these samples were obtained from provided their written informed consent to participate in this study, and the Ethical Review Committee of the WHO Collaborating Center for Research in Human Production authorized by the Shanghai Municipal Government approved this study and the consent procedure. Total RNA was extracted from 101 HCC primary tumors and adjacent non-tumor tissues. Additionally, we primarily employed the tumor-nude-metastasis (TNM) to determine the histopathological grade of HCC, and two categories were classified (low grade, I–II; high grade, III–IV).

### Cell lines and cell culture

Huh-7, SK-Hep-1, SNU-449 and HEK-293T were obtained from American Type Culture Collection (ATCC). Cell line has been authenticated by ATCC based on morphology, karyotyping and PCR assays. MHCC- 97L, MHCC-97H and HCC-LM3 were obtained from and authenticated by Liver Cancer Institute (Zhongshan Hospital, China). All cells were cultured at 37°C with a 5% CO_2_ atmosphere in DMEM supplemented with 10% fetal bovine serum, 100 U/ml penicillin, and 100 μg/ml streptomycin.

### Antibodies, plasmids and other reagents

Antibodies against SNAIL and E-cadherin were purchased from Cell Signaling Technology (Danvers, MA, USA). Antibodies against SLC39A6 were purchased from ProteinTech Group (Chicago, IL, USA). The antibody against β-actin was purchased from Sigma (St. Louis, MO, USA). The GAPDH antibody was purchased from Kangcheng (Shanghai, China). The miR-192 mimic and inhibitor were synthesized by RiboBio (Guangzhou, China). The *SLC39A6* ORF was amplified from the Huh-7 cell cDNA and subcloned into pWPXL. The wild type and mutant 3′UTRs of *SLC39A6* were synthesized from GENEWIZ (Suzhou, China) and subcloned into the firefly luciferase reporter. Small interfering RNAs (siRNAs) targeting *SLC39A6* and negative control siRNA were ordered from RiboBio (Guangzhou, China). miRNA probes were purchased from Life Technologies (Shanghai, China).

### Lentiviral vector construction, packaging and infection

The entire coding sequence of *SLC39A6* and pre-miR-192 were amplified and cloned into the pWPXL vector, which was obtained from Addgene (http://www.addgene.org). Lentivirus production and transduction were performed according to instructions supplied by Addgene. Packaging and infection of lentivirus were performed as previously described [23].

### Luciferase reporter system

HEK-293T cells cultured in a 96-well plate were co-transfected with 10 nM NC or miR-192 mimic, 2 ng pRL-TK (Promega, Madison, WI, USA) and 10 ng firefly luciferase reporter containing the wildtype or mutant 3′UTR of *SLC39A6*. Transfections were performed in triplicate and repeated in three independent experiments. Forty-eight hours after transfection, luciferase activity was analyzed using a Dual-Luciferase Reporter Assay System (Promega, Madison, WI, USA) and a microplate fluorescence reader (BioTek).

### Quantitative real-time PCR

Total RNA was extracted from the tumor tissues. First-strand cDNA synthesis and amplification were performed using Reverse Transcription Reagents (TaKaRa, Dalian, China) according to the manufacturer's instructions. cDNA templates were mixed with SYBR Green Premix with ROX (TaKaRa, Dalian, China) to perform quantitative PCR reactions. β-actin was used as an endogenous control for the mRNA levels. miRNAs were synthesized by TaqMan probes as reverse transcription primers. Reverse transcription products were mixed with SYBR Green Premix with ROX (TaKaRa, Dalian, China) to perform quantitative PCR reactions. Details regarding the primer sequences are provided in Supplementary Table 2.

### Western blot analysis

For western blot analysis, the protein concentrations were determined by the Bradford assay. Cell lysates were separated by SDS-PAGE and transferred to polyvinylidenedifluoride membranes. The membranes were blocked with 5% non-fat milk in TBST and incubated with specific primary antibodies. The detection of protein complexes was performed using SuperSignal West Pico chemiluminescent substrate (Thermo Scientific) and imaged using an ImageQuant LAS4000 biomolecular imager (General Electric Company).

### Cell proliferation assays

Cells were seeded in 96-well plates at a density of 2,000 cells per well and incubated with complete medium. Culturing medium was removed, and aliquots (10 μl) from the Cell Counting Kit-8 (CCK-8, Dojindo, Kumamoto, Japan) plus 90 μl complete medium were added to the wells and incubated for 2 hours. After incubation, the absorbance was measured at 450 nm. Each measurement was performed in triplicate, and the experiments were repeated at least twice.

### Transwell migration and invasion assays

Cell migration assays were performed using 6.5-mm Transwell chambers (8 μm pore size, BD). Invasive abilities were examined using chambers pre-coated with Matrigel. Cells were seeded in Transwell chambers, and serum was used as the chemo-attractant in the lower chamber. Then, 16 to 24 hours later, the cells that moved to the basal side of the membrane were fixed, stained with crystal violet, visualized and imaged using a CKX41 microscope (Olympus) at 200 × magnification. Images of three random fields from three replicate wells were counted.

### Animal experiments and histological analysis

Five- to six-week-old male congenitally immuno-deficient BALB/c nude mice were maintained under specific pathogen-free (SPF) conditions. The mice were manipulated and housed according to protocols approved by the Shanghai Medical Experimental Animal Care Commission. Huh-7-miR-192 and Huh-7-vector cells (2 × 10^6^ per mouse) were injected into the livers of nude mice. Six weeks later, the mice were euthanized, and the livers were removed and processed for standard histological study. For histological analysis, the livers were fixed in 10% formalin. Then, the fixed samples were embedded in paraffin, and three non-sequential serial sections were obtained from each animal. The sections were stained with hematoxylin and eosin (H & E) and analyzed for the presence of metastases.

### Statistics

The statistical analyses and graphical depiction of data were performed using GraphPad Prism 5.0. The results are presented as the mean ± SEM and were evaluated by Student's *t*-test (two-tailed; *P* < 0.05 was considered significant) unless otherwise specified (paired *t*-test, Pearson's correlation). Additionally, certain statistical calculations were performed using SPSS (Statistical Package for the Social Sciences) version 19.0 for Windows. The chi-square (χ^2^) test was used to evaluate the correlation between metastasis incidence and miR-192 expression in animal model. Kaplan-Meier methods were used for survival analysis. The Cox hazard regression model was used in univariate and multivariate analyses. *P* < 0.05 was considered significant.

## SUPPLEMENTARY MATERIALS FIGURES AND TABLES


