# Supplementary material for: miR-192, a prognostic indicator, targets the SLC39A6/SNAIL pathway to reduce tumor metastasis in human hepatocellular carcinoma
Source: Oncotarget. 2015 Dec 14;7(3):2672–83. doi: 10.18632/oncotarget.6603 (PMC4823063; doi:10.18632/oncotarget.6603)
Supplement: Supplementary file 1 [file oncotarget-07-2672-s001.pdf]

miR-192, a prognostic indicator, targets the SLC39A6/SNAIL pathway to reduce tumor metastasis in human hepatocellular carcinoma

Supplementary Materials

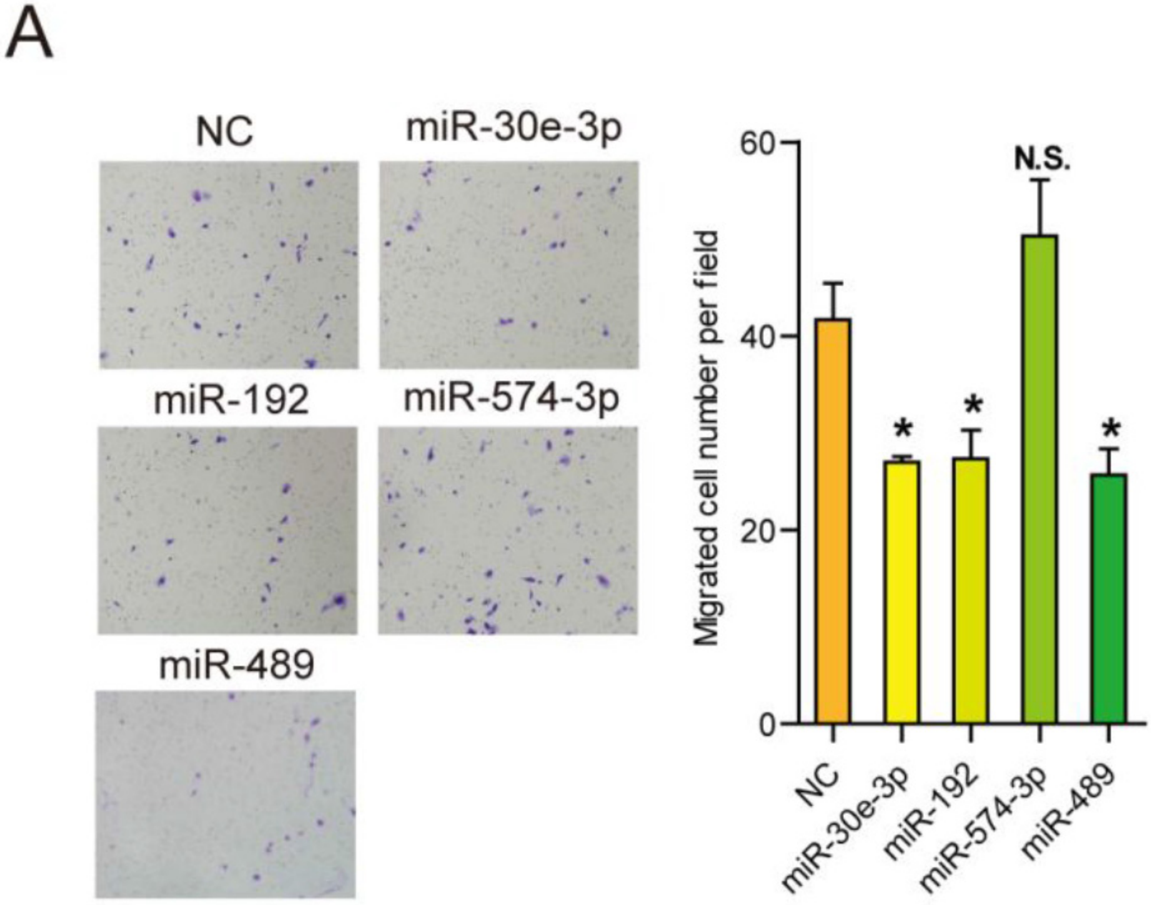

Supplementary Figure S1: (**A**) Migration assays for four candidate miRNAs in Huh-7 cells Representative results of the trans-well migration assays for the impact of miR–30e–3p, miR–574–3p, miR–489 and miR–192 on the migratory abilities of Huh–7 cells (unpaired Student's t-test, mean ± s.e.m; *$P < 0.05$; **$P < 0.01$; ***$P < 0.001$).

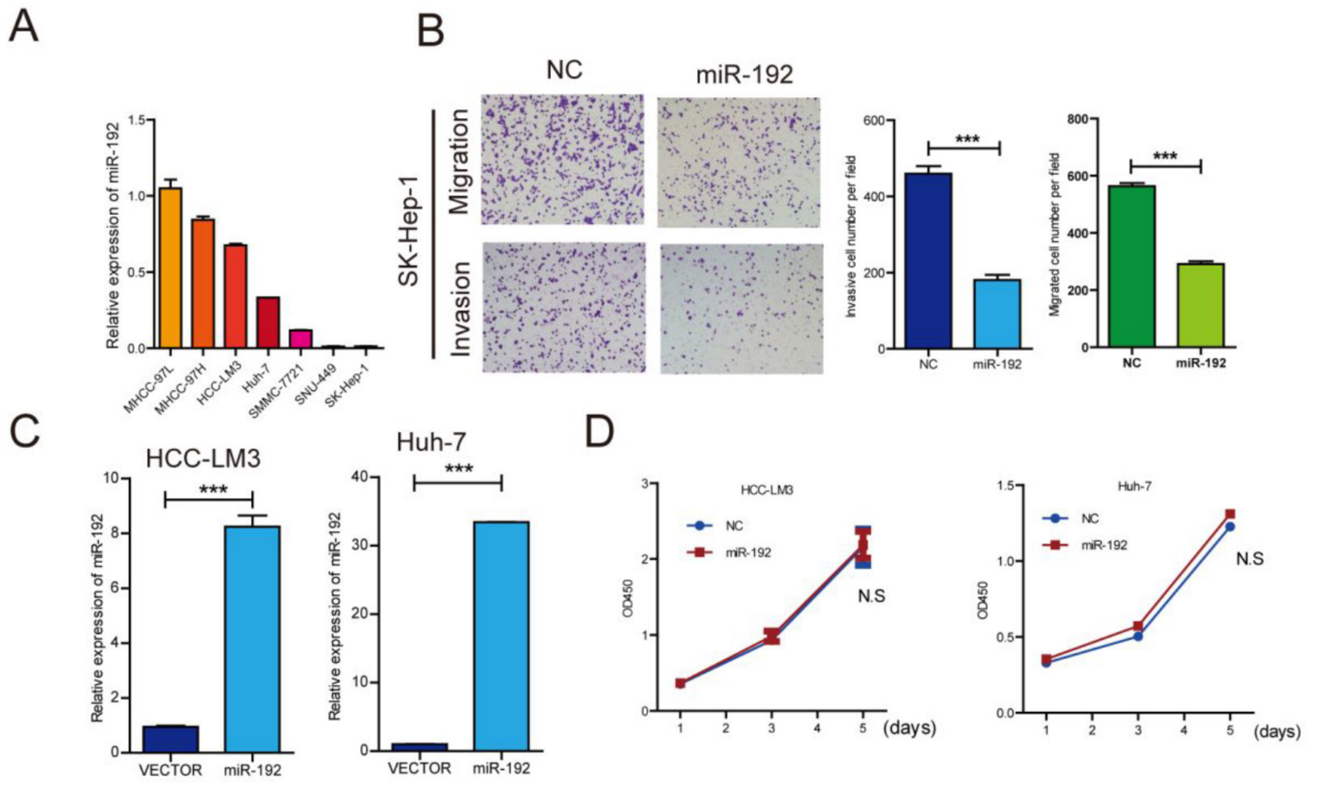

Supplementary Figure S2: (**A**) Determination of miR-192 expression in seven HCC cell lines by quantitative real-time PCR (q-PCR). (**B**) Representative results of the trans-well migration and invasion assays for the impact of miR-192 on the migratory and invasive abilities of SK-Hep-1 cells (unpaired Student's *t*-test, mean ± s.e.m; **P* < 0.05; ***P* < 0.01; ****P* < 0.001). (**C**) Determination of miR-192 overexpression in stable cell lines by q-PCR. (**D**) Representative results of CCK8 assay for the impact of miR-192 on cell proliferation.

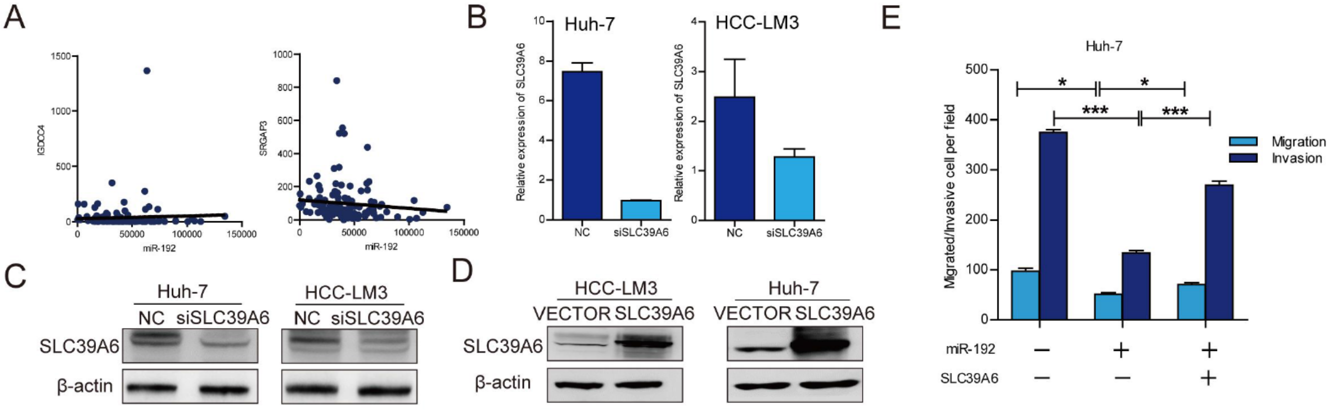

Supplementary Figure 3: (**A**) Correlation analysis of miR-192 expression and IGDCC4 and SRGAP3 expression in 130 HCC tissues in TCGA database. The correlation was analyzed with a two-tailed Pearson Correlation Test. (**B** and **C**) Determination of SLC39A6 siRNA interference efficiency by q-PCR and Western Blot. (**D**) Western Blot analysis of SLC39A6 overexpression in HCC-LM3 and Huh-7 cells. (**E**) Transwell migration and invasion assays for the SLC39A6-rescued migratory and invasive abilities of Huh-7 overexpressing cells (unpaired Student's *t*-test, mean ± SEM; **P* < 0.05; ***P* < 0.01; ****P* < 0.001).

Supplementary Table 1: 27 miRNA expression status in microarray and TCGA dataset

ID	Microarray signal			TCGA data				
	MHCC-97L	MHCC-97H	HCC-LM3	*p* value	FDR	Log_mean_tumor	Log_mean_normal	Log_mean_diffrence
**Upregulation**								
hsa-miR-1231	3.259767	4.507083	5.097651	N.A.				
hsa-miR-125b-2-3p	3.294928	4.494297	5.743264	5.62E-09	7.90E–08	4.4380102	5.7931617	–1.355152
hsa-miR-3940-5p	7.984017	8.658625	9.129696	N.A.				
hsa-miR-4505	5.177657	5.92159	6.469844	N.A.				
hsa-miR-4508	8.122631	8.736217	9.20644	N.A.				
hsa-miR-4665-5p	4.368652	4.956302	5.909731	N.A.				
hsa-miR-638	9.11582	9.733932	10.21006	0.32232522	0.42167511	0.0074345	0	0.007434
hsa-miR-149-3p	7.604098	8.312204	8.613513	0.43633807	0.53361813	0.0878833	0.1149926	–0.027109
hsa-miR-1908	7.636138	8.373727	8.758545	0.82931265	0.8750228	0.0631244	0.0563211	0.006803
hsa-miR-3180	2.266128	4.096238	4.642979	0.21055623	0.31701725	0.0478204	0.0208678	0.026953
hsa-miR-3619-5p	3.234108	4.077185	4.602365	N.A.				
hsa-miR-3621	4.76202	5.54164	6.093533	0.32232522	0.42167511	0.0048686	0	0.004869
hsa-miR-4417	2.585245	3.658118	4.014865	N.A.				
hsa-miR-4488	9.074427	9.77784	10.13928	N.A.				
hsa-miR-4688	1.861713	3.078107	3.514257	N.A.				
hsa-miR-4695-5p	6.444691	7.11744	7.198678	N.A.				
hsa-miR-663	7.151887	7.941307	8.568822	0.00049857	0.00205113	0.2073916	0.5062657	–0.298874
hsa-miR-762	8.079802	8.886806	9.321171	N.A.				
hsa-miR-99a-5p	8.105789	9.318857	10.02874	8.15E-10	1.53E–08	9.0406556	10.670173	–1.629518
**Downregulaion**								
hsa-miR-489	6.935359	5.977414	5.733166	0.01387672	0.03546692	0.1534533	0.294876	–0.141423
hsa-miR-194-3p	5.316932	4.546129	3.824315	2.38E-05	0.00013118	6.3630204	7.3517453	–0.988725
hsa-miR-200a-3p	2.587555	1.27415	1.035936	3.39E-07	3.03E–06	3.5288858	5.6545797	–2.125694
hsa-miR-1301	6.412939	5.429835	5.477927	3.35E-06	2.29E–05	3.1537757	2.3180283	0.835747
hsa-miR-675-5p	5.855566	4.950084	4.553838	0.24476579	0.35595398	0.4419509	0.6096352	–0.167684
hsa-miR-30e-3p	2.479794	1.623412	1.942846	3.67E-09	5.47E–08	13.149134	13.925196	–0.776063
hsa-miR-192	6.529882	5.688693	5.689155	0.00207722	0.00705954	14.89122	15.628211	–0.736991
hsa-miR-574-3p	3.712222	3.492945	3.124977	1.74E-06	1.27E–05	7.0410491	7.651349	–0.6103

Supplementary Table 2: List of primers for q-PCR

Gene	Forward primer(5′-3′)	Reverse primer(5′-3′)
SLC39A6	TGAAATCCCTCCAAAGAC	CCACTCAAAGTCCCAACG
IGDCC4	AATACGGTTTGGGAAAGGAA	GACATGGCTCTGGTTGTGC
SRGAP3	CTGGATTCCCGAAGTGAC	AGCTGGTGATAACGCATG
DLG5	CTCCTTCCGCTCAGATGC	CTTTCTTTGCTTTGGCTTTT
TYMS	TCTGCCAGTTCTATGTGGTG	GAAAGGTCTGGGTTCTCG
C9orf100	GGAGGTTTGTACGGCTTCA	GTGCTGGTCATTCTTCTGTTTC
NSF	TGCTTCCCGAGTATTTCC	GCCTCTGATTCTCCCACA
C4orf46	CCTGAGGAGTTGCAGGTT	GTGGCTTCAAGAAGTTTGGT
KIF20B	GAATAAAGCACCAGAGTG	TTCCCAGAGTCAATAAAG
LPAR4	AGTTGTTGGGTTTATCATTC	AGGGCATACAAGAAGAGG
